# Accounting for risk in valuing forest carbon offsets

**DOI:** 10.1186/1750-0680-4-1

**Published:** 2009-01-16

**Authors:** Matthew D Hurteau, Bruce A Hungate, George W Koch

**Affiliations:** 1 Department of Biological Sciences and Merriam-Powell Center for Environmental Research, PO Box 6077, Flagstaff, AZ 86011, USA

## Abstract

**Background:**

Forests can sequester carbon dioxide, thereby reducing atmospheric concentrations and slowing global warming. In the U.S., forest carbon stocks have increased as a result of regrowth following land abandonment and in-growth due to fire suppression, and they currently sequester approximately 10% of annual US emissions. This ecosystem service is recognized in greenhouse gas protocols and cap-and-trade mechanisms, yet forest carbon is valued equally regardless of forest type, an approach that fails to account for risk of carbon loss from disturbance.

**Results:**

Here we show that incorporating wildfire risk reduces the value of forest carbon depending on the location and condition of the forest. There is a general trend of decreasing risk-scaled forest carbon value moving from the northern toward the southern continental U.S.

**Conclusion:**

Because disturbance is a major ecological factor influencing long-term carbon storage and is often sensitive to human management, carbon trading mechanisms should account for the reduction in value associated with disturbance risk.

## Background

Terrestrial ecosystems sequestered approximately 30% of anthropogenic emissions from 2000–2006 [1], and the potential to manage the carbon sink strength of forested systems in particular has garnered much attention from cap-and-trade mechanisms. Regrowth due to land abandonment and in-growth due to fire suppression have resulted in an increase in U.S forest carbon stocks [2], sequestering approximately 10% of annual U.S. emissions [3]. In addition to reforestation, increasing carbon density and reducing emissions from disturbances (fire, insect outbreaks) are strategies for using forests to slow the rise of atmospheric carbon dioxide (CO_2_) [4]. Compared to these "upside" perspectives, risks have largely been ignored when considering investment in forest carbon management. The recent increase in frequency of large and severe fires due to past fire suppression and ongoing climate change [5,6] illustrates one such risk [7]. From 2002–2006, carbon emissions resulting from wildfire were equivalent to four to six percent of annual anthropogenic emissions [8]. Under current carbon accounting mechanisms, all forest carbon offset projects are equivalent provided they sequester more carbon than business-as-usual ("additionality") and that the additional carbon is maintained in the forest for a pre-determined period of time ("permanence"). However, permanence does not incorporate risk of carbon loss from disturbance. Some forests are at greater risk than others, giving them greater potential for rapidly releasing large quantities of stored carbon to the atmosphere as CO_2_. Here we show that the value of forest carbon declines by as much as 99% when the risk of loss due to wildfire is considered.

Under current carbon accounting mechanisms, carbon permanence is typically defined as 100 years (e.g. California Climate Action Registry, Forest Project Protocol 2007), and does not incorporate risk analysis. A largely qualitative non-permanence risk analysis has been proposed for maintaining a 'buffer pool' of carbon as a hedge against loss [9]. However, without a quantitative risk assessment, this method does not allow for efficient sizing of buffer pools.

## Results

We use the fire regime condition class departure index (FRCC_DEP) and mean fire return interval (mFRI) data products developed for the LANDFIRE project [10] to discount the market value of forest carbon as a function of the risk of loss due to wildfire. The FRCC_DEP is an index of departure of the current vegetation condition from the historical range of variability for the system. It ranges from no departure (0) to complete departure (1) depending on fire severity type probabilities. Mean fire return interval represents the average time between fire years at a given location.

We use mFRI as the probability of a fire event occurring during a specified time period and FRCC_DEP to estimate the potential carbon loss given a fire occurrence. The discounted market value of a ton of carbon (*V*_*d*_) is estimated as:

$$V_{d}=\left\{ \begin{array}{l} V_{c}\left[1-F\left(1-\frac{M}{P}\right)\right],\;when\;M<P\\ V_{c},\;when\;M\geq P \end{array}\right.$$

where *V*_*c *_represents the current market value for a ton of carbon, *M *represents mFRI in years, *F *represents FRCC_DEP, and *P *represents permanence, which is the length of time in years a ton of forest carbon must be present to meet trading mechanism protocols.

**Figure 1:**
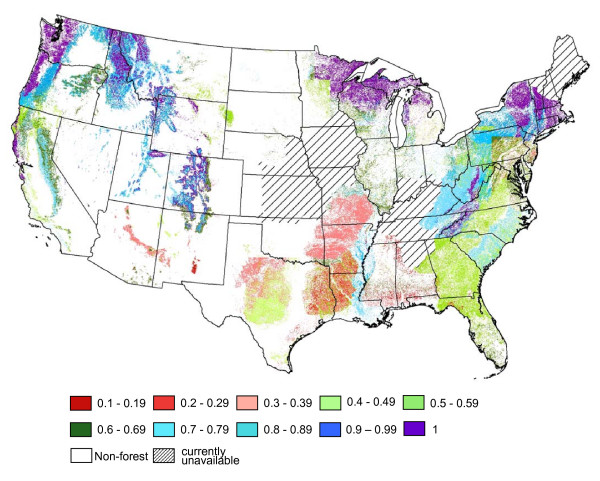
**Continental U.S. risk-scaled carbon value map**. Map of the continental U.S. showing average relative carbon value, $1-F\left(1-\frac{M}{P}\right)$, by forest type. Multiplying the average relative carbon value on the map by the market value of carbon determines the risk scaled value of the forest carbon for a given forest type. For example, a value of 0.4 equates to a risk scaled value equal to 40% of the market value.

Using a permanence value of 100 years results in risk-scaled values ranging from 100% of market value (*M *≥ *P*) to 1% of market value (*M *= 1, *F *= 1). In the continental U.S., discounted market values vary by forest type and tend to decrease on a north to south gradient (Figure 1). Using the available 30 m resolution LANDFIRE data products, the risk of carbon loss resulting from wildfire can be estimated for specific locations allowing for site-specific, risk-incorporated market valuation.

## Discussion

With this method, the value of forest carbon becomes sensitive to the risk of its loss. For example, a unit of carbon in fire-prone ponderosa pine forests of the U.S. Southwest is worth only 30% of that in redwood forest in California. This is due to the greater deviation from the Historic Range of Variability of the ponderosa pine (*F *= 0.82), coupled with the more frequent occurrence of fire (*M *= 16 years).

Stainback and Alavalapati [7] suggest that in even-aged plantation forestry, risk from natural disturbance reduces the incentive that a carbon market would provide to landowners for increasing rotation age and thus carbon stocks. Previous research has suggested that for forests historically characterized by frequent, low severity fire, thinning the forest can reduce the risk of carbon loss from wildfire [11,12]. Thus, FRCC_DEP can be altered, making this carbon valuation method robust to site-specific management actions, providing incentive in terms of increased carbon market value for landowners to engage in high severity fire risk reduction measures.

Substitution of FRCC and mFRI with the appropriate metrics would allow application of this approach to adjusting market value of forest carbon due to risks of other disturbances such as forest insect outbreaks and hurricanes [13,14]. This methodology provides economic incentive for managing forest systems based on their individual ecologies or departure from sustainable conditions.

We suggest that further research is needed in two areas to provide a more accurate assessment of the risk of carbon loss from wildfire. The mean fire return interval LANDFIRE data product was developed from simulation modeling that was in part parameterized by fire history studies and local expert knowledge [15]. Thus, it is limited by the availability of fire history studies and would benefit from increased efforts to reconstruct fire history for larger geographic areas. The second area in need of further research is the relationship between FRCC_DEP and direct carbon loss from fire. While FRCC_DEP does not translate directly into potential carbon loss resulting from a wildfire event, it does capture deviation of current conditions from historic conditions in terms of fire size, frequency, severity, or intensity [15]. Hardy et al. [16] indicate that as departure from historic conditions increases, increasing levels of mechanical thinning and the application of prescribed fire will be necessary to restore the system. Previous research has indicated that without fuels reduction treatments, such as mechanical thinning and prescribed fire, wildfire events result in higher direct carbon emissions [11,12].

## Methods

The Landscape Fire and Resource Management Planning Tools Project, known as LANDFIRE, was established in part to develop landscape-scale geospatial data products to support fire and fuels planning [10]. As part of LANDFIRE, researchers developed the Fire Regime Condition Class Departure Index (FRCC_DEP). FRCC_DEP is based on work by [16-18] that developed a three class departure metric to quantify the deviation of current conditions from historic conditions, where class one is within the historical range of variability, class two represents a departure of more than one fire return interval, and class three represents a departure by multiple fire return intervals. Classes two and three represent moderate and substantial changes, respectively, in fire size, frequency, severity, or intensity [16]. FRCC_DEP is a comparison between current conditions and historical reference conditions [19] and was constructed by calculating the approximation error between current vegetation data vectors and historical simulated vegetation conditions [20]. The FRCC departure index is based on the concept of historical range of variability (HRV), ranging from zero (no departure from HRV) to one (complete departure) [19]. Keane et al. [19] define HRV as the fluctuation in natural processes and ecological characteristics prior to Euro-American settlement of the western U.S. for the time period between 1600 and 1900 AD. The pre-Euro-American time period represents a period prior to effective fire suppression efforts [21]. Calculation of the FRCC departure index involved establishing HRV using the LANDSUMv4 succession model, which models succession as a deterministic process and disturbance events as stochastic processes [22]. The probability of a fire event in the LANDSUMv4 model is a function of the influence of weather on fire ignition and spread, a scaling factor determined by average fire and patch size, fire return interval, and time since fire [15,19]. The severity of the fire event is a function of the potential vegetation type and succession class [22]. The probabilities associated with each fire severity type (surface, mixed-severity, and stand replacing fire) are determined from simulations in LANDSUMv4 [19,21]. Fire severity and average fire return intervals used to parameterize disturbance modelling in LANDSUMv4 were obtained from literature searches and consultation with local experts [15]. The occurrence of a fire event alters the successional pathway of the vegetation community at the location of the event [21]. The HRV simulations in LANDSUMv4 are used to quantify the mean Fire Return Interval (mFRI), which is the average number of years between fire events at a given location. The mFRI is calculated by dividing the length in years of the simulation by the number of times fire occurs at a specific location [22].

We subtract the mFRI divided by the permanence value from one and then multiply by the FRCC_DEP to obtain the contribution of one fire return interval to the FRCC_DEP over the permanence period and scale the FRCC_DEP by the contribution of one fire return interval to FRCC_DEP. Subtracting the scaled FRCC_DEP from one results in the discount coefficient.

**Figure 2:**
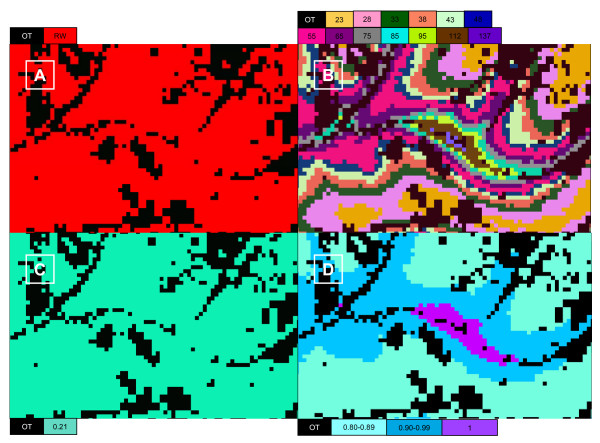
**Data layer processing**. An example of data layer processing for a tract of Redwood forest. Each panel is representative of the same land area and is 2.2 km by 1.4 km in size. Panel A is the image classified to represent either redwood forest (RW) or other forest type (OT). Panel B is the mean fire return interval in years for each 30 meter pixel from the LANDFIRE data product. The mean fire return intervals in the panel range from 23 to 137 years. Panel C is the fire regime condition class departure index value from the LANDFIRE data product. Panel D is the relative carbon value, $1-F\left(1-\frac{M}{P}\right)$, for each pixel of redwood forest, incorporating the mean fire return interval and fire regime condition class departure data products. Multiplying the relative carbon value by the market value of carbon determines the risk scaled value of carbon for each pixel.

We extracted the data inputs necessary for our equation from the FRCC departure index, mean fire return interval, and existing vegetation type data layers (Figure 2). Using the raster math tool in ArcGIS (ESRI), we implemented the equation to generate Figure 1, showing the average relative carbon value by forest type for continental U.S.

## Competing interests

The authors declare that they have no competing interests.

## Authors' contributions

MH developed the C valuation method in collaboration with BH and GK. MH conducted the spatial analysis and all authors contributed to the writing. All authors have read and approved the final version of the manuscript.
